# Appropriate Data Quality Checks Improve the Reliability of Values Predicted from Milk Mid-Infrared Spectra

**DOI:** 10.3390/ani11020533

**Published:** 2021-02-18

**Authors:** Lei Zhang, Chunfang Li, Frédéric Dehareng, Clément Grelet, Frédéric Colinet, Nicolas Gengler, Yves Brostaux, Hélène Soyeurt

**Affiliations:** 1 TERRA Teaching and Research Centre, University of Liège—Gembloux Agro-Bio Tech, 5030 Gembloux, Belgium; lei.zhang@doct.uliege.be (L.Z.); frederic.colinet@uliege.be (F.C.); nicolas.gengler@uliege.be (N.G.); y.brostaux@uliege.be (Y.B.); 2 Hebei Livestock Breeding Station, Shijiazhuang 050000, China; chunfangli0521@126.com; 3 Key Lab of Agricultural Animal Genetics, Breeding and Reproduction of Ministry of Education, Huazhong Agricultural University, Wuhan 430070, China; 4 Valorisation of Agricultural Products Department, Walloon Agricultural Research Centre, 5030 Gembloux, Belgium; f.dehareng@cra.wallonie.be (F.D.); c.grelet@cra.wallonie.be (C.G.)

**Keywords:** milk-component prediction, mid-infrared spectrum, Mahalanobis distance, quality-assurance system, Holstein cow

## Abstract

**Simple Summary:**

There is a growing interest in using milk mid-infrared (MIR) spectrometry to obtain new phenotypes to assist in the complex management of dairy farms. These predictive values can be erroneous for many reasons, even if the prediction equations used are accurate. Unfortunately, there is no quality protocol routinely implemented to detect those abnormal predictive values in the database recorded by dairy herd improvement (DHI) organizations, except for fat and protein contents. However, for financial and practical reasons, it is unfeasible to adapt the quality protocol commonly used in milk laboratories to improve the accuracy of those traits. So, this study proposes three different statistical methods that would be easy to implement by DHI organizations to detect abnormal values and limit the spectral extrapolation in order to improve the accuracy of MIR-based predictive values.

**Abstract:**

The use of abnormal milk mid-infrared (MIR) spectrum strongly affects prediction quality, even if the prediction equations used are accurate. So, this record must be detected after or before the prediction process to avoid erroneous spectral extrapolation or the use of poor-quality spectral data by dairy herd improvement (DHI) organizations. For financial or practical reasons, adapting the quality protocol currently used to improve the accuracy of fat and protein contents is unfeasible. This study proposed three different statistical methods that would be easy to implement by DHI organizations to solve this issue: the deletion of 1% of the extreme high and low predictive values (M1), the deletion of records based on the Global-H (GH) distance (M2), and the deletion of records based on the absolute fat residual value (M3). Additionally, the combinations of these three methods were investigated. A total of 346,818 milk samples were analyzed by MIR spectrometry to predict the contents of fat, protein, and fatty acids. Then, the same traits were also predicted externally using their corresponded standardized MIR spectra. The interest in cleaning procedures was assessed by estimating the root mean square differences (RMSDs) between those internal and external predicted phenotypes. All methods allowed for a decrease in the RMSD, with a gain ranging from 0.32% to 41.39%. Based on the obtained results, the “M1 and M2” combination should be preferred to be more parsimonious in the data loss, as it had the higher ratio of RMSD gain to data loss. This method deleted the records based on the 2% extreme predictions and a GH threshold set at 5. However, to ensure the lowest RMSD, the “M2 or M3” combination, considering a GH threshold of 5 and an absolute fat residual difference set at 0.30 g/dL of milk, was the most relevant. Both combinations involved M2 confirming the high interest of calculating the GH distance for all samples to predict. However, if it is impossible to estimate the GH distance due to a lack of relevant information to compute this statistical parameter, the obtained results recommended the use of M1 combined with M3. The limitation used in M3 must be adapted by the DHI, as this will depend on the spectral data and the equation used. The methodology proposed in this study can be generalized for other MIR-based phenotypes.

## 1. Introduction

Since the 1980s, milk contents like fat and protein have been predicted using milk mid-infrared (MIR) spectrometry. Thanks to the high accuracy of the predictive models used, those phenotypes are included in the equation that defines the milk price paid to farmers for their production. Due to the direct economic link between those MIR predictions and farm profitability, most milk laboratories follow a quality-assurance procedure proposed by the Internal Dairy Federation during what is most commonly called the “ring test”. During this test, identical sets of modified milk samples (i.e., often 10 milk samples having a large variation of fat content) are measured using MIR spectrometers in the different laboratories. The predictive values are then compared to the contents given by certified reference chemical-measurement methods. This allows for inter- and intra-laboratory comparisons (i.e., machine differences or operators), as well as the definition of the slope and the bias corrections used to more accurately calibrate the fat and protein predictions given by the manufacturer’s equation after the prediction process. Moreover, if the bias and slope corrections for the considering trait are too strong, the ring test alerts the laboratory that an adjustment is needed, or a maintenance of the spectrometer is required. A procedure is also implemented to check the daily (short-term) stability of the instrument between two ring tests. The ring test has a cost due to the creation and sending of milk samples, the reference chemical analysis, and the laboratory comparisons. But this cost is the price to ensure the best predictive values and accuracy for the fat and protein phenotypes predicted by MIR. In parallel, as the milk laboratories involved in the milk analysis for the milk payment and the routine recording of dairy cows performance are the same, the ring test is a great opportunity for dairy herd improvement (DHI) organizations to ensure a high quality of the fat and protein phenotypes used in their breeding selection programs.

Farms are requiring an increasing amount of highly skilled labor and techniques to handle complex cow management strategies [1]. Fast and low-cost phenotypes related to milk production, animal husbandry, animal health, and reproduction are therefore currently required for dairy farming [2,3,4]. This explains why these phenotypes are increasingly being predicted with a fluctuating accuracy by milk MIR spectrometry [4]: fatty acids composition [5,6], protein composition [6], mineral contents [7], milk acidity [8], energy status of cow [9], nitrogen-utility efficiency [10], cow fertility [11,12], methane emissions [13], and detection of milk adulteration [14]. This means that all of these phenotypes are derived from the same raw data: the MIR spectrum. For this reason, an increasing number of DHI organizations record the milk MIR spectrum of each tested cow in their database in order to perform a specific prediction externally using their own prediction equations. For instance, the Walloon Breeding Association (Awé, Ciney, Belgium) was the first to realize this recording. However, compared to the predicted contents of fat and protein, no quality protocol is currently implemented in milk laboratories (except the alert for machine maintenance) or in DHI organizations for these new MIR traits. The improvement of the ring-test procedure to take into account these traits is sometimes unfeasible, as it is impossible to quantify them using chemical analysis of milk (i.e., methane, energy balance). Moreover, for traits measurable directly in milk, the high cost of the reference chemical analysis will dramatically increase the final cost of the ring test, potentially impacting its large use by milk laboratories or DHI organizations, especially because no direct economic return for the farmer exists for those traits currently. So, an alternative low-cost and quick quality protocol must be defined.

To achieve this objective, it is important to highlight the different factors influencing the final accuracy of an MIR prediction (i.e., predictive values). First, the prediction equations itself can provide inaccurate predictive values due to the variability of the dataset used to build it. From a dataset containing reference data and their corresponding milk MIR spectra, prediction equations can be developed and easily applied to new milk spectra to predict the phenotypes of interest. However, the performance of this modeling can fluctuate with the conditions of application. The stability of the prediction equation, more often called robustness, is worth considering. For decades, there is no standard definition of robustness [15]. It is certain that a high accuracy (i.e., a low root mean square error (RMSE)) coupled with a low RMSE variability under various conditions contribute to robust prediction equations. Grelet et al. [16] reported that the inclusion of the variability related to milk spectra, breeds, herds, and diets can notably improve the model’s robustness. Thomas and Ge [17] also concluded that the validity of a prediction equation depends partly on the representativeness and the structure of the calibration set used. However, for practical and financial reasons, it is impossible to include all samples as needed to cover the entire variability of field data [18]. This could potentially affect the prediction accuracy for new samples. This means that even if we have a high-quality model, the obtained predictions can be erroneous due to the spectral extrapolation, as the sample variability is not taken into account in the calibration set. Consequently, knowing the calibration set used to develop a prediction equation is of interest to limit the predictive values of samples that are out of the variability covered by the set. Second, a poor-quality predictive value can be obtained due to the use of poor-quality spectral data. Unfortunately, there is no consensus regarding the best strategies to be implemented routinely to address both of these issues. As mentioned previously, a strategy based on the analysis of traits using a reference chemical analysis is not feasible for financial or practical reasons. However, a quality protocol based on statistical procedures applied directly or indirectly on the spectral data massively available for DHI organizations could be relevant and cost-efficient.

Indeed, multivariate distance can be calculated between the spectrum to be predicted and those used in the calibration set in order to limit spectral extrapolation. The Mahalanobis distance [19] (also called H distance) is preferable to the Euclidean distance, as the H distance takes the variability and the internal correlations of samples into consideration [20,21]. However, the Global H (GH) distance is preferred in practice because it is not affected by the number of variables used to calculate this distance. The GH distance is defined as the ratio of the squared H distance to the number of variables used [22,23]. A GH value equal to 3 was proposed to eliminate potential outliers [22]. However, in the case of a multivariate normal model, less than 99% of the population would fall within the boundary of 3, and the optimal limit of the GH value actually depends on the size of the calibration set, the number of variables used, and the alpha risk limit adopted [24]. The high quality of information that can be provided by the calculation of such a distance could be implemented by milk laboratories or DHI organizations in the future.

To identify potential outliers for a particular trait, experimenters often use the mean ± 3 times the standard deviation if the trait is normally distributed (alpha = 0.3%). If the GH distance is not available, since the MIR traits are often normally distributed and due to the high quantity of information available by DHI organizations or milk laboratories, a cleaning based on these easy-to-compute position statistical parameters is relevant. Moreover, these potential outliers can be related to a spectral extrapolation during the prediction process or the use of poor-quality spectral data. This last case could also be detected using the content of fat given directly by the spectrometer, as this predictive value is routinely adjusted for bias and slope by milk laboratories during the ring test. Due to this correction and the high accuracy of the prediction equation used, this predicted content can therefore be assumed to be a control value. The estimation of a large error between this control value and the value predicted using an external prediction equation (i.e., without bias and slope corrections) could highlight the presence of abnormal spectra due to analytical issues or a wrong association between the sample and the data. Recently, Dale et al. [25] proposed the cleaning of an MIR prediction dataset using a threshold of 2% of the relative fat error.

In conclusion, since an increasing number of phenotypes is predicted from milk MIR spectra externally of the spectrometers, a definition of a quality procedure is needed to ensure the reliability of these phenotypes. To achieve a realistic context, this study deals with DHI data collected during routine milk recording. Milk fatty acids and protein contents were used in this study to illustrate the interest in the cleaning of statistical approaches previously detailed. However, these proposed procedures can be generalized to any other MIR phenotypes. So, three data quality checks were tested: the deletion of 1% of extreme high and low predicted values (Method 1), the deletion of records based on the spectral GH distance (Method 2), and the deletion of records based on the absolute residual value between the predicted and control milk fat contents (Method 3). Additionally, combinations of these three methods were investigated.

## 2. Materials and Methods

### 2.1. Data

A total of 397,131 milk records were collected from 49,522 Holstein dairy cows belonging to 279 herds from 2018 to 2019 in Shijiazhuang (Hebei province, China). All milk samples were analyzed on four Bentley FTS instruments (Bentley, MN, USA) to predict the contents (g/dL of milk) of fat and protein that were then corrected using the slope and bias estimated from ring tests. The spectrometer also predicted the contents of monounsaturated fatty acids (MFA), unsaturated fatty acids (UFA), and saturated fatty acids (SFA) (g/dL of milk) from the generated MIR spectral data using predictive models established by the manufacturer.

All MIR spectra were standardized following the procedure detailed by Grelet et al. [26] based on piecewise regressions. Briefly, spectral standardization consists of comparing the spectral data obtained by different milk laboratories and instruments using the same milk samples in order to estimate standardization coefficients needed to ensure a high reproducibility of spectral data between laboratories. For this study, the standardization procedure was performed two times, in December 2018 and February 2019. The standardization coefficients that were obtained closest to the test date were applied to the recorded spectral data. The average interval between the spectral measurement and the standardization was 117 days. Based on the standard requirements proposed by the International Committee for Animal Recording (ICAR) [27], records out of the range of 1.5–9.0% for fat and 1.0–7.0% for protein were deleted. Only records having a day in milk between 5 and 365 were kept. The average lactation number ranged from 1 to 13 with a mean value of 2. Finally, the cleaned dataset contained 346,818 records collected from 49,522 Holstein cows. The average number of records per cow was 7 ± 11.

The contents of fat, protein, MFA, UFA, and SFA predicted by the spectrometer were compared to those predicted from recorded standardized spectra using external prediction equations, for which basic information is listed in Table 1.

### 2.2. Data-Cleaning Techniques

A total of three different methods were tested in this study to clean the MIR-based predicted phenotypes. Their relevancy was assessed by estimating the improvement of the relationship between the manufacturer’s and predicted values, measured by calculating the RMSD using the following formula:

(1) $$\mathrm{RMSD}=\sqrt{\sum_{i=1}^{n}{\left(EXT_{trait}-INT_{trait}\right)}^{2}/n}$$

where *n* is the total number of observations of the considered trait, *EXT_trait_* is the content predicted externally using the prediction equation, and *INT_trait_* is the manufacturer’s prediction.

As the MIR predictive values were nearly normally distributed based on their estimated skewness and kurtosis values (Table 2), and as the dataset was large and representative of the studied dairy population (i.e., more than 300,000 records), the first data-cleaning method consisted of removing extreme MIR external predicted values based on the observed 1% and 99% quartile values (Method 1). The fixed thresholds used to clean the dataset for all predicted traits are given in Table 3.

The second data-cleaning technique tested in this study was based on the calculation of the GH distance between a considered sample spectrum and the average spectrum calculated from the calibration set used to build the external prediction equation (Method 2). This was done to observe if this spectral record was not too distant from the calibration set. This distance was calculated for all spectral records by first calculating the H distance and then by standardizing this distance using the number of variables used [28]. As many spectral data points are highly correlated with each other, a reduction of the spectral dimensionality was needed to inverse the (co)variance matrix required to calculate the H distance. So, a principal component analysis was performed on the first-derived standardized spectra in the calibration set. A first derivation using a gap of 5 was applied to the standardized spectra in order to correct the baseline drift. Then, the obtained eigenvectors were applied to the recorded spectral data in order to resume their spectral information into new variables called principal components (PCs). The number of PCs was fixed to cover 95% of the calibration spectral variability. The GH value between a recorded sample spectrum and the calibration average spectrum was calculated using the following formula:

(2) $$\mathrm{GH}=\left({\left(\bar{x}-\bar{\mu }\right)}^{T}S^{-1}\left(\bar{x}-\bar{\mu }\right)\right)/n\mathrm{PC}$$

where $\bar{x}$ is the PC scores of the spectrum to be predicted, $\bar{\mu }$ is the mean of PC scores estimated from the calibration set, S corresponds to the (co)variance matrix between PC scores estimated from the calibration spectra, and nPC is the number of principal components used. Other formulas exist to calculate the GH distance by introducing factors related to the number of samples used in the calibration set (e.g., *n*/*n*−1 [29]), but we decided to use a simplified formula, as the number of samples used in the calibration set was large. Therefore, the additional factor was close to 1. As the squared differences between internally and externally predicted values were not normally distributed, Kruskal–Wallis nonparametric tests and pairwise two-sample Wilcoxon comparisons were used to assess the significance of observed squared differences from GH thresholds ranging from 1 to 10. Moreover, the correlation coefficient between GH distances and those squared differences was also estimated in order to observe the strength of their linear relationship.

The third method tested in this study was derived from the procedure proposed by Dale et al. [25]. As explained in the Introduction section, milk samples with known contents of fat and protein are analyzed using MIR spectrometry by many milk laboratories during the ring tests. This allows the estimation of bias and slope needed to correct the prediction of fat and protein contents done using milk MIR spectrometry. Therefore, those corrected predictions can be assumed to be similar to the reference values measured by a reference chemical analysis. This means that the calculation of the absolute difference between this assumed control fat content and the content predicted externally using a prediction equation could be relevant for cleaning the prediction dataset. As the fat content predicted externally is not corrected by applying the slope and bias, this quantification is directly influenced by the quality of the spectral data. So, the working hypothesis of Method 3 is that the absolute fat residual difference could be used to detect poor-quality spectra due to an instrumental issue, an analytical issue, or a wrong association between the sample and the data. Four absolute fat residual thresholds (0.08, 0.10, 0.30, and 0.40 g/dL of milk) were tested, and the obtained squared differences between the manufacturer’s and externally predicted contents were compared using Kruskal–Wallis nonparametric tests and pairwise two-sample Wilcoxon comparisons.

Finally, using a similar statistical approach, the interest in combining these cleaning methods was also studied.

## 3. Results

### 3.1. Comparison between Manufacturer’s and Externally Predicted Phenotypes

The average values of externally predicted fat, protein, and fatty acids contents were higher than those obtained directly from the Bentley spectrometers (Table 2). The maximum values of the externally predicted phenotypes were also higher for all traits except for SFA. However, the coefficient of variation (CV) stayed within the same range for all studied traits, as did the standard deviation (SD) values. Moreover, the relationships between the studied traits were good, as high correlation coefficients were obtained between the manufacturer’s and externally predicted phenotypes (i.e., ≥0.94; Table 2), even if they were inferior to the square root of the cross-validation R² listed in Table 1. However, even if the error was higher, the prediction performance was better for the traits predicted with a higher accuracy by MIR spectrometry like fat, protein, and SFA.

### 3.2. Deletion of Extreme Predicted Phenotypes (Method 1)

Method 1 consisted of cleaning the raw dataset by deleting records that were out of the range estimated using the externally predicted values observed at the 1% and 99% quartiles as thresholds. The impact of this deletion on the dataset is shown in Table 3. The loss of data in each trait was always around 2%, and the SD of externally predicted traits decreased due to the deletion of extreme values. Method 1 allowed an RMSD decrease of around 1% for fat and protein traits, and around 2% for fatty acids traits (Table 3). This was expected for UFA and MFA, as these traits presented a large range of variation (Table 2).

### 3.3. GH-Based Data-Cleaning (Method 2)

The estimated GH distances varied between traits (Table 4) due to the use of different calibration sets to build the external prediction models (Table 1). These calibration sets differed based on their size and their spectral representativeness. The eigenvectors estimated using the PC analysis were different between traits. Consequently, the PC scores used to estimate the GH distances were also different. A large majority of samples (i.e., more than 79%) had a GH lower than or equal to 3. A higher proportion of samples had a GH greater than 5 for MFA and UFA. This means that the models used to predict those fatty acids were less adapted for some Chinese samples even if, on average, the GH distances stayed below 3 (Table 4). In other words, the presence of extremely high GH distances highlighted the presence in the dataset of samples having a spectrum largely different than the ones used in the calibration set.

The correlation values between the GH distance and the squared differences between the manufacturer’s and externally predicted traits were moderately positive and ranged between 0.35 and 0.48 (Table 4), except for protein, for which a correlation coefficient of 0.07 was obtained. This low correlation could signify that the prediction of this milk component was less affected by the spectral extrapolation.

As there was no agreement regarding the GH threshold, we decided to study the prediction relationship between the manufacturer’s and externally predicted traits after applying different GH thresholds ranging from 1 to 10. Data loss for the different studied traits ranged from around 1%, when a GH threshold was set at 10, to around 72% when set at 1. When the GH threshold was lower than 4, the data loss for each trait was higher than 10%. As the squared differences between the manufacturer’s and externally predicted traits were not normally distributed, Kruskal–Wallis nonparametric tests and pairwise two-sample Wilcoxon comparisons were performed to compare those values. No differences (*p* > 0.05) of squared differences for all studied traits were observed between groups based on a GH limitation of between 7 and 8, or between 9 and 10 (except MFA and UFA). Globally, the average squared differences decreased significantly (*p* < 0.05), from GH limitation at 7 to GH limitation at 1 for all studied traits.

If we applied a GH threshold equal to 5, which corresponds to an acceptable data loss (less than 10%), a significant decrease of the root mean square difference, and a value recognized in the literature as an appropriate threshold to detect outliers, the decrease of RMSD ranged from 1.29% for protein to 8.88% for fat (Table 5). The low gain observed for the protein content was related to the low correlation that existed between the GH distance and the squared differences (Table 4). Indeed, there was a correlation value of 0.75 between the gain and the correlation values between GH distance and the squared differences. However, the number of discarded data also had an influence, but its intensity was weak (correlation of 0.15 between the gain and the percentage of data loss). The highest RMSD was observed for the fat and fatty acids contents. It is also interesting to note that the SD values for externally predicted traits were closer to those estimated using the manufacturer’s traits shown in Table 2. By applying a GH threshold set at 5, the RMSD gains estimated for all studied traits were higher than the ones observed when applying Method 1.

### 3.4. Data Cleaning Based on the Absolute Fat Residual Limit (Method 3)

The strategy in Method 3 consisted of applying a limitation based on the absolute value of the difference between the fat content externally predicted and the one quantified by the Bentley spectrometer and then corrected using the slope and bias estimated after ring tests (i.e., assumed control fat content). This strategy should allow detecting spectra with poor quality. Indeed, the squared differences for fat contents were positively correlated with all studied traits (0.13 for protein and 0.40 for the remaining studied traits). This moderate relationship could support the potential interest in using absolute fat differences to detect abnormal spectra. Compared to the study of Dale et al. [25], in which data loss was equal to 30% due to the threshold (≤2% relative error fat value), the current study proposed to use the absolute residual between the predicted and assumed control fat contents to clean the dataset. Several limitations were tested, and they ranged from 0.08 to 0.40 g of fat per dL of milk. The data loss was equal to 55.93%, 47.31%, 8.88%, and 3.47% for the thresholds set at 0.08, 0.10, 0.30, and 0.40 g/dL of milk, respectively. The significance of the squared differences between internally and externally predicted phenotypes were assessed based on Kruskal–Wallis nonparametric tests and pairwise two-sample Wilcoxon comparisons. From these results, we can conclude that the stricter the absolute fat residual limitation, the greater the data loss and the better the RMSD gain, except for protein. More specifically, in most cases, the squared differences decreased (*p* < 0.05) from the group cleaned using a limit set to 0.40 g/dL of milk to the group with a limitation of 0.30 g/dL of milk. The loss of data using this last fat residual limitation was less than 10% (Table 6).

When a threshold of 0.30 g/dL of milk was applied to clean the dataset, the RMSD gain ranged from 1.49% for protein to 38.82% for fat. The higher improvement observed for fat is obvious because this trait was directly involved in the cleaning process proposed in Method 3. However, even if high correlation values exist between fat and fatty acids, the RMSD gain for fatty acids was not as high as the one observed for fat. The lowest gain was reached by the protein content. However, even if low, this improvement was better than the ones observed based on Method 1 and Method 2. This was also confirmed for the other studied traits except for MFA, for which Method 2 provided a slightly better improvement.

### 3.5. Comparison of the Three Tested Data-Cleaning Methods

In this study, we tested three different techniques to clean an MIR-predicted dataset, and we concluded that all of them allowed for a decrease of the squared differences between the manufacturer’s and externally predicted phenotypes. Method 3 had the highest RMSD gain except for MFA. However, these methods can be combined during a quality procedure. Highlighting the best combination of methods will depend on the aim. In this study, we proposed two different aims. The first was to highlight the method offering the highest RMSD gain. The second was based on a compromise by selecting the model that offered a high RMSD gain and a low data loss. To achieve this, we calculated the ratio between the gain and the data loss expressed both in percentage. All results about method combinations are listed in Table 7.

The method combinations offering the highest RMSD gain were “M2 or M3” for fat and UFA and “M1 or M2 or M3” for the remaining traits. So, the combination of Methods M2 and M3 seems to be crucial. This combination allowed the detection of samples for which the prediction could be hazardous due to a spectral extrapolation, as well as the use of spectral data with poor quality. By comparison with “M2 and M3” or “M1 and M2 and M3”, it seems important to discard not only a record that fulfils the condition of M2 or M3, as the RMSD gain was higher.

The method combinations offering the best compromise between the number of samples discarded (i.e., N loss in Table 7) and the RMSD gain were: “M1 and M2” (i.e., higher gain:loss). For protein, the combination “M1 and M2 and M3” provided a slightly better result. So, the combination “M1 and M2” seems to be important. This means that to achieve the best gain:loss ratio, we need to conserve samples for which the prediction values were not extreme and the GH distance was lower than 5 to avoid spectral extrapolation.

## 4. Discussion

As mentioned in the Introduction, an increasing number of phenotypes are being predicted from milk MIR spectrometry to develop management and breeding tools for dairy farmers. Unfortunately, for financial and practical issues, it is not possible for milk laboratories or DHI organizations to routinely analyze a set of reference samples. Therefore, adapting the ring tests developed to ensure the quality of the MIR predictive values of fat and protein contents is not feasible. So, this study proposed to test different statistical approaches easily implementable in practice at a low cost and a large scale to clean the DHI database related to MIR-based predictive values. To achieve this objective, three methods were tested and combined. By predicting the MIR phenotypes on a large scale, the obtained dataset can be assumed as representative of the studied dairy cow population and, therefore, useful to identify extreme values. As the majority of MIR-predicted traits are normally distributed, the thresholds to consider a record as an outlier can be based on the 1% and 99% quartiles. This was the principle applied in Method 1 to clean the DHI dataset. Even if this method presents the advantages of being easy to implement and robust thanks to the definition of the threshold for a large database based on individual cow measurements, this method automatically forced the deletion of around 2% of extreme records without knowing the reasons, potentially leading to a decrease in the existing variability for the studied trait. However, knowing the origin of the extreme values could be interesting to confirm the outlier status. So, incorrect MIR predictive values can be obtained due a spectral extrapolation related to a prediction process applied to samples for which the spectral variability was not taken into account in the calibration set used to build the predictive model. To solve this issue, the calculation of the GH distance between the average calibration spectrum and the spectrum of the sample to predict is useful. This is the basic of Method 2. The greater the GH distance, the more the prediction can be the result of spectral extrapolation. However, this method has the inconvenient of requiring the (co)variance matrix of the calibration set and is not able to identify predictive values made on poor-quality spectra (i.e., samples for which an important slope and bias correction was applied on fat and protein predictions). So, a third method was proposed in this study to achieve this objective that was based on the absolute residual fat difference. If the difference estimated between the corrected fat prediction content and the one predicted directly from the spectral data was high, the probability that the spectrum has a poor quality was high. In conclusion, this means that Method 1 is a rough method to clean a database compared to Method 2 and Method 3 which focus more on a potential origin of prediction uncertainty.

Ideally, to compare those methods, we need a set of reference values. However, for financial and practical reasons, this was unrealistic. Moreover, when the milk analysis is available, its cost limits the size of the dataset, which is often no more representative of the studied dairy cow population. For instance, this is the case for the calibration set used to build MIR predictive models. Indeed, in order to increase the variability needed in the calibration set to develop a robust model, the samples are not selected randomly, but their selection is controlled to ensure a good covering of the existing variability of the studied trait. Consequently, in this study, we decided to use the records directly provided by the spectrometers: the contents of fat, protein, and the major groups of milk fatty acids. Fat and protein predictions were assumed to be control values, as these values were corrected based on the slope and bias estimated by the milk laboratory after ring tests. Moreover, it is known that the prediction equations to quantify these traits are very accurate (Table 1). For fatty acids, the accuracy of the prediction equations used in the spectrometers was unknown. However, it is known that this accuracy is higher for the equations predicting SFA compared to the ones quantifying MFA or UFA (Table 1). Fortunately, the FA predictive values provided by the used Bentley spectrometers were within the expected range. Indeed, although few previous Chinese investigations have been conducted into the analysis of milk-fat composition from Holstein cows based on a large-scale MIR spectral database, the contents obtained in the current study were within the expected range for Chinese Holstein cows [30,31]. Using the same technology to predict the fatty acids contents, Bastin et al. [32] reported average contents of MIR-predicted MFA, SFA, and UFA equal to 1.13, 2.79, and 1.31 g/dL of milk produced by Holstein cows. Those contents were slightly higher than the values obtained in the current study. On the other hand, the contents of MFA, SFA, and UFA obtained in the current study were lower than those reported by Soyeurt et al. [5] (i.e., 1.44, 2.95, and 1.65 g/dL of milk) and Fleming et al. [33] (1.00, 2.97, and 1.16 g/dL of milk from Holstein, Brown Swiss, and Jersey cows, respectively).

The fat, protein, SFA, MFA, and UFA were predicted both by the Bentley spectrometers and external prediction equations (Table 1). Even if the prediction accuracy of Bentley spectrometer to predict the contents of those traits was unknown, good relationships between those predictions were observed, with correlation coefficients higher or equal to 0.94 (Table 2). The higher squared differences between those predictions compared to the cross-validation RMSE, a traditional parameter to measure the accuracy of a prediction model [34], can be attributed to several reasons. First, the calibration dataset used to build the external prediction models did not contain samples collected on the studied Chinese dairy cow population. Therefore, the spectral variability of some samples were not taken into account, leading to an uncertainty in the prediction due to the spectral extrapolation. Second, the spectra were standardized to be expressed on a FOSS basis in order to apply the available external prediction equations. This could introduce an additional uncertainty. Third, even if the contents of fat and protein were assumed to be a control value, all records were not analyzed using reference chemical analysis. The bias and slope correction cannot perfectly correct all predictions. However, the aim of this study was not to validate the prediction equations used in the spectrometer or applied externally. Therefore, the error shift was not so problematic, as this study focused on the changes of the RMSD value following the cleaning procedures. Even if the RMSD values listed in Table 2 were higher than the RMSE values mentioned in Table 1, the high correlations between the predicted traits, as well as their similar CV (Table 2), are sufficient to confirm the interest in using those data to conduct the proposed research and realize their representativeness due to the large individual data acquisition. The higher SD observed for the traits predicted externally compared to the ones provided by the spectrometer were due to the presence of outliers in the dataset. Indeed, Næs et al. [35] noted that if the new sample to be predicted was beyond the capacity of calibration models, the accuracy was impaired accordingly.

For all traits, the cleaning methods decreased the RMSD and allowed the SD values for the externally predicted phenotypes to be closer to the ones estimated from the manufacturer’s predictive values (Table 3, Table 5 and Table 6). These SD decreases confirmed that a part of the variability observed in the raw data was related to several outlier records. However, all proposed cleaning methods did not discard the same records. For instance, less than 1% of records were considered as outliers for the three methods used. About 1% of the records had M1 and M2 or M1 and M3 in common. Close to 3% of records had M2 and M3 in common. Method 1 was a rough cleaning procedure that deleted 2% of the extreme predictions. Therefore, this method can keep outliers in the dataset if more than 2% of records are outliers. Following the considered traits and from the results listed in Table 7, Method 2 had between 25% and 46% of its discarded samples in common with Method 3. It was less than 25% for Method 1.

This moderate relationship between Method 2 and Method 3 can be easily explained. Method 2 discarded the samples out of the spectral range present in the calibration set. A GH limit of 3 was proposed by Mark and Tunnell [36], as three standard deviations from the center of a group was considered to be the boundary. In the case of a multivariate model, a threshold of 3 may be unjustifiable for the dataset and the number of wavelengths used [24]. A threshold of around 4 was proposed by Ritchie et al. [37] based on the distribution of samples. In our study, this GH threshold was set at 5 to limit the data losses (i.e., lower than 6% except for MFA and UFA; Table 5) and to significantly decrease the RMSD. Method 3 detected spectra data with poor quality. Therefore, samples with poor spectral data must have had a high GH distance. So, the samples discarded by Method 2 and Method 3 certainly had a very poor spectral quality. The existence of these abnormal or extreme spectra could be related to different factors that can be associated, for instance, with instrument failure, incorrect milk sampling, an analytical issue, or poor milk conservation between the sampling and analysis period. The remaining samples discarded by Method 3 were related to samples for which the spectral data was only far from the one present in the calibration, but the spectral data did not seem to have a poor quality. For instance, these abnormal or extreme spectra could be related to specific cows showing a unique spectral pattern and therefore special milk composition. This could have a potential negative impact on a breeding program, as those cows could potentially be interesting. However, this was not observed in this study. The average loss of records for a specific cow was equal to 0.91 (i.e., less than 1 record per cow). The most probable reason could be related to a different farm management and context. Indeed, it is known that the milk composition can be influenced by many factors related to the animal or to the farming system. In this last issue, the feeding system has a major impact on the milk-fat composition. The external prediction equations used in this study were not developed using Chinese samples. Therefore, the milk spectral data could be not fully considered. However, a large part of the spectra used in this study had a GH lower than 3 (Table 4), which means that those spectra were within the spectral range of the calibration set, even if the studied records were not included in the prediction equations. This also indirectly showed that globally, the milk composition of studied samples was similar to the samples included in the calibration set, which were collected in Europe. However, even if only 5 to 9% of samples were discarded based on a GH threshold set at 5 (Table 5), samples in the dataset with an uncovered spectral variability remained. This was especially true for the models that predicted the contents of MFA and UFA, which are known to be influenced by feeding, but also the heat stress and the body condition score [38,39,40]. This finding suggests an interest in specifically collecting these kinds of samples to improve the robustness of the fatty acids prediction models. However, the finding could not conclude whether the accuracy could be improved as the gas-chromatographic fatty-acid profiles were not available for these samples, which had a GH distance out of the desired range. The percentage of samples discarded using a GH threshold was similar to previous results. For instance, based on drug samples, Morozova et al. [41] found 77.3% of spectra with a H distance (also GH distance, as GH is obtained by dividing the squared H distance by the number of variables) less than 3, 18.2% of spectra with a H distance between 3 and 4, and 4.5% of spectra greater than 4. Based on a GH threshold equal to 3, Soyeurt et al. [42] deleted 3.9% of the raw data. This is lower than the data loss observed in this study using the same threshold, which ranged from 12.48% to 20.27%. This could be related to the fact that the calibration sets used to build the prediction equations did not consider the records included in the current study, leading to a potentially higher number of records considered as outliers, but related to a spectral milk variability that was not considered in the prediction equations. However, as mentioned previously, the main part of the data had a GH lower than 3.

Concerning the samples discarded by Method 2, only between 25% and 46% of those samples were in common with Method 3 (results derived from Table 7). This means that the majority of the samples discarded by Method 3 were kept by Method 2. As the RMSD gain was the highest for nearly all traits (Table 7), we can assume that this cleaning, even if stricter, provided a beneficial effect. These samples presented spectral data considered as poor-quality, but their spectral profile was not extreme, as the GH distance was lower than 5. The average GH distance for those samples was equal to 2.20, with minimum and maximum values of 0 and 5.00, respectively. Consequently, as Method 2 and Method 3 reflected different outlier origins, it was logical to observe an additional improvement of RMSD gain by combining these methods with the “or” logical disjunction (Table 7).

For protein, SFA, and MFA, a slight improvement was obtained by adding the Method 1 using an “or” logical disjunction to Method 2 and Method 3 (Table 7). This means that Method 1 and Method 3 were not able to detect fully all outliers. However, the improvement was low compared to the “M2 or M3” combination, leading to the highlighting of a poor contribution of Method 1. However, the cleaning procedures having the highest gain:loss ratios were combinations involving Methods 1 and 2 using an “and” logical disjunction (Table 7). Only for the protein content, adding Method 3 in this combination improved slightly the gain:loss ratio. Method 1 and Method 2 both dealt with extreme values, the first based on the predictions, and the second based on spectral data. The selection of a cleaning procedure based on this ratio represents an optimization between the data loss and the RMSD gain. The “M1 and M2” combination allowed the detecting of samples with an extreme predictions and a GH distance higher than 5. This means that samples out of the spectral range covered by the set used to build the prediction models, but having a reasonable prediction, were kept in the dataset. Indeed, the samples kept in Method 1 but not in Method 2 had an average GH ranging from 7.02 to 9.08, with a minimum value of 5.10 and a maximum value of 113.00 for all studied traits. In other words, this implies that we accepted the spectral extrapolation during the prediction process if the prediction is between a realistic range. This working hypothesis is acceptable, as the correlation values between GH distance and the squared residuals were moderate for fat and fatty acids and low for protein (Table 4). The positive correlation found in this study is in agreement with the findings of Whitfield et al. [24], who reported that the H distance was directly proportional to the absolute residuals between the predicted and reference values. The GH-based cleaning deleted less than 1% of raw records, which is largely lower than the “M2 or M3” combination, which discarded from 11 to 15% (Table 7).

Depending on the aim of the deletion of potential outlier samples (lower RMSD gain or higher gain:loss ratio), the selected cleaning method was not the same. However, Method 2 is the common denominator. This reveals a high interest in measuring the GH distance for all samples to predict. Practically, this means that simultaneously providing the GH distance of the spectrum to be predicted with the prediction could be relevant to indirectly inform customers about the potential accuracy of the given phenotype. However, the GH calculation requires the mean PC spectrum, the eigenvectors, and the (co)variance matrix between calibration PC scores. This information could be provided by the manufacturer or the creator of the equation. Currently, they are reluctant to provide this information because they do not want to communicate the calibration set. However, the milk laboratories and DHI organizations do not need the dataset, but only the average PC spectrum, the eigenvectors, and the covariance matrix, which should not be confidential. Some international institutions like the International Committee for Animal Recording (ICAR) or the International Dairy Federation (IDF) could pressure the model’s providers to obtain this information. However, if it is impossible to find, a cleaning based on the deletion of 1% of extreme high and low predicted values estimated from a large spectral database is still of interest, combined with Method 3 (Table 7).

The protein content was the trait that was less affected by the data cleaning. Indeed, the RMSD gain ranged from 0.32 to 2.54% (Table 7). This low improvement could be explained by the fact that this trait was less impacted by the spectral extrapolation. Of course, the correlation values between the GH distance and the squared differences between the manufacturer’s and externally predicted phenotypes were low (Table 4). Moreover, this trait also presents a low variability compared to the other studied traits (i.e., CV of 12% vs. 30–40% for the other traits; Table 2). It could be supposed that Method 3 as based on fat content, which is correlated with fatty acids, could be more profitable for these traits compared to the protein. But Method 3 considered alone also offered the best improvement in the protein content, highlighting an interest in using this cleaning process for traits that are less correlated with the fat content as well.

The choice of the fat content to develop the cleaning criteria of Method 3 was related to the fact that this trait is always adjusted for the slope and bias by the milk laboratory conducting routine ring tests, and presents a natural large variability (Table 2). So, in the DHI database, this trait can be assumed to be closer to the real reference value. By comparing this assumed control value to the one predicted using the external prediction equation, it was possible to detect spectral abnormal values that can lead to erroneous MIR predictive values. So, from the results obtained in this study, a fat limitation of 0.30 g/dL of milk seems to be preferable in order to increase the chance to gain accurate predictions without losing much data. However, the limitation must be adapted to the spectral database used, as this will depend on the spectral data and the equation used.

## 5. Conclusions

This study confirms that spectral outliers are generated within the framework of DHI, as the current corrections for the slope and bias used in the milk laboratories to ensure high prediction quality for fat and protein contents had no effect on the spectral data. Moreover, some spectral outliers that were different from the spectra constituting the calibration dataset were also identified. So, there is a need to define a quality procedure for MIR phenotypes, especially as the amount of traits predicted by this technology is growing rapidly. Three methods were tested and combined in this study. To be more parsimonious in the data loss, the results recommended to prefer the “M1 and M2” combination, which involved the deletion of the 2% extreme predictions + samples with a GH higher than 5. However, to ensure the lowest squared differences between manufacturer’s and externally predicted phenotypes, the “M2 or M3” combination, in which M3 consisted of deleting samples with an absolute fat residual higher than 0.30 g/dL of milk, must be applied on the DHI dataset. Both of those combinations involved Method 2 confirming the high interest of calculating the GH distance for all samples to predict. However, if it is impossible to estimate the GH distance due to a lack of relevant information to compute this statistical parameter, we recommend the use of Method 1 combined with Method 3 (i.e., M1 or M3). The limitation used in Method 3, which was set to 0.3 g/dL of milk, must be adapted to the spectral database used, as this will depend on the spectral data and the equation used. The methodology proposed in this study can be applied to find the best threshold for the considered database, and could be implemented on any MIR-based phenotypes.

## Figures and Tables

**Table 1:** Prediction performances of the external equations used to predict the contents of five milk components.

Trait (g/dL of Milk)	N ^1^	Mean ± SD ^2^	Cross-Validation	
			RMSE *	R^2^
Fat	1799	3.93 ± 1.00	0.0086	0.9999
Protein	4305	3.36 ± 0.41	0.0200	0.9976
Monounsaturated fatty acids	1793	1.08 ± 0.34	0.0581	0.9705
Saturated fatty acids	1790	2.69 ± 0.74	0.0719	0.9904
Unsaturated fatty acids	1788	1.24 ± 0.37	0.0648	0.9698

* RMSE = root mean square error. ^1^ N is the number of samples used in the calibration set. ^2^ SD is the standard deviation.

**Table 2:** Descriptive statistics of manufacturer’s and externally predicted phenotypes (g/dL of milk) and their relationships.

N = 346,818	Parameters	Fat	Protein	MFA ^1^	SFA ^1^	UFA ^1^
Manufacturer’s values	Mean	3.93	3.41	0.85	2.58	0.92
	SD ^2^	1.10	0.42	0.32	0.76	0.38
	CV ^3^	27.99	12.32	37.65	29.46	41.30
	Minimum	1.01	1.01	0.04	0.10	0.01
	Maximum	8.99	6.99	4.61	8.00	5.19
	Skewness	0.65	0.40	1.59	0.79	1.44
	Kurtosis	1.52	2.62	6.00	2.12	5.07
Externally predicted values	Mean	3.94	3.53	1.13	2.62	1.28
	SD	1.10	0.47	0.42	0.75	0.46
	CV	27.92	13.31	37.17	28.63	35.94
	Minimum	0.72	0.52	−5.62	0.13	−6.44
	Maximum	9.92	7.10	5.26	7.32	5.66
	Skewness	0.69	0.28	1.22	0.72	1.12
	Kurtosis	1.63	1.88	5.56	1.80	5.39
Prediction relationship ^4^	RMSD	0.173	0.187	0.327	0.199	0.397
	r	0.99	0.95	0.94	0.97	0.94

^1^ MFA = monounsaturated fatty acids; SFA = saturated fatty acids; UFA = unsaturated fatty acids. ^2^ SD = standard deviation. ^3^ CV = coefficient of variation. ^4^ Root mean square difference (RMSD) and correlation value (r) between manufacturer’s and externally predicted values.

**Table 3:** Characteristics of the dataset obtained after a cleaning using the externally predicted values observed at the 1% and 99% quartiles as thresholds (Method 1).

Traits	Threshold		N	Data loss (%)	g/dL of milk			Gain_RMSD_^2^ (%)
	1%	99%			Mean	SD	RMSD ^1^	
Fat	1.59	7.35	339,909	1.99	3.93	0.99	0.17	1.29
Protein	2.49	4.78	339,941	1.98	3.52	0.42	0.185	0.97
MFA ^3^	0.35	2.51	339,708	2.05	1.12	0.36	0.319	2.62
SFA ^3^	1.06	4.92	339,852	2.00	2.61	0.68	0.195	1.95
UFA ^3^	0.4	2.78	339,892	2.00	1.27	0.40	0.389	2.16

^1^ RMSD = root mean square difference between manufacturer’s and externally predicted phenotypes. ^2^ Gain = difference expressed in % between RMSD estimated before and after cleaning with Method 1. ^3^ MFA = monounsaturated fatty acids; SFA = saturated fatty acids; UFA = unsaturated fatty acids.

**Table 4:** Descriptive statistics of Global-H (GH) distances and their correlation coefficient (r) with the squared differences between the manufacturer’s and externally predicted traits (e^2^).

Traits	GH Distance			GH ≤ 3	3 < GH ≤ 5	GH > 5	r
	Mean	SD	Max	(%)	(%)	(%)	(GH,e^2^)
Fat	1.84	2.66	182.5	86.61	8.27	5.12	0.35
Protein	1.90	4.11	182.6	86.36	7.99	5.65	0.07
Monounsaturated FAs ^1^	2.37	3.23	211.3	79.73	11.42	8.85	0.48
Saturated FAs	1.78	2.48	170.3	87.52	7.84	4.64	0.47
Unsaturated FAs	2.37	3.23	209.7	79.70	11.46	8.85	0.47

^1^ FAs = fatty acids.

**Table 5:** Characteristics of the dataset obtained after a cleaning based on a Global-H (GH) distance set at 5 (Method 2).

Traits	N	Data Loss (%)	g/dL of Milk			Gain_RMSD_^2^ (%)
			Mean	SD	RMSD ^1^	
Fat	329,064	5.12	3.89	1.01	0.159	8.88
Protein	327,230	5.65	3.52	0.42	0.185	1.29
Monounsaturated fatty acids	316,128	8.85	1.1	0.37	0.303	7.79
Saturated fatty acids	330,739	4.64	2.59	0.70	0.188	5.97
Unsaturated fatty acids	316,128	8.85	1.25	0.40	0.376	5.72

^1^ RMSD = root mean square difference between the manufacturer’s and externally predicted values. ^2^ Gain = difference expressed in % between RMSD estimated before and after cleaning using Method 2.

**Table 6:** Characteristics of the dataset obtained after a cleaning based on an absolute fat residual limitation set at 0.30 g/dL of milk (Method 3).

N = 316,025	g/dL of Milk			Gain_RMSD_^2^ (%)
	Mean	SD	RMSD ^1^	
Fat	3.88	1.06	0.125	38.82
Protein	3.53	0.46	0.184	1.49
Monounsaturated fatty acids	1.1	0.38	0.304	7.52
Saturated fatty acids	2.58	0.73	0.185	7.64
Unsaturated fatty acids	1.24	0.42	0.373	6.35

^1^ RMSD = root mean square difference between the manufacturer’s and externally predicted values. ^2^ Gain = difference expressed in % between RMSD estimated before and after cleaning using Method 2.

**Table 7:** Interest of using the three studied data-cleaning methods and their combinations to clean the raw dataset.

Traits	Parameters	M1 ^1^	M2 ^1^	M3 ^1^	M1 and M2	M1 and M3	M2 and M3	M1 and M2 and M3	M1 or M2	M1 or M3	M2 or M3	M1 or M2 or M3
Fat	Gain_RMSD_^3^ (%)	1.29	8.88	38.82	38.82	1.70	8.39	1.40	8.88	38.77	41.39	41.08
	N loss ^4^ (%)	1.99	5.12	8.88	0.76	0.33	2.30	0.26	6.35	10.54	11.69	12.85
	Gain:loss ^5^	0.65	1.73	4.37	51.23	5.09	3.64	5.48	1.40	3.68	3.54	3.20
Protein	Gain_RMSD_ (%)	0.97	1.29	1.49	1.49	0.35	0.66	0.32	1.60	2.21	2.22	2.54
	N loss (%)	1.98	5.65	8.88	1.41	0.36	1.42	0.29	6.22	10.50	13.11	13.61
	Gain:loss	0.49	0.23	0.17	1.06	0.97	0.47	1.12	0.26	0.21	0.17	0.19
MFA ^2^	Gain_RMSD_ (%)	2.62	7.79	7.52	7.52	1.74	4.69	1.62	7.96	8.77	11.36	11.42
	N loss (%)	2.05	8.85	8.88	0.93	0.57	2.98	0.48	9.97	10.36	14.75	15.78
	Gain:loss	1.28	0.88	0.85	8.07	3.07	1.58	3.38	0.80	0.85	0.77	0.72
SFA ^2^	Gain_RMSD_ (%)	1.95	5.97	7.64	7.64	2.27	3.34	0.65	6.63	9.29	10.99	11.60
	N loss (%)	2.01	4.64	8.88	0.58	1.02	2.13	0.15	6.07	10.63	11.38	12.71
	Gain:loss	0.97	1.29	0.86	13.22	2.22	1.57	4.20	1.09	0.87	0.97	0.91
UFA ^2^	Gain_RMSD_ (%)	2.16	5.72	6.35	6.35	1.57	3.82	1.48	5.74	7.18	8.74	8.67
	N loss (%)	2.00	8.85	8.88	0.92	0.56	2.95	0.47	9.92	10.32	14.77	15.76
	Gain:loss	1.08	0.65	0.72	6.87	2.82	1.29	3.17	0.58	0.70	0.59	0.55

^1^ M1 = Method 1; M2 = Method 2 with a GH limitation set at 5; M3 = Method 3 with an absolute residual fat difference set at 0.30 g/dL of milk. ^2^ MFA = monounsaturated fatty acids; SFA = saturated fatty acids; UFA = unsaturated fatty acids. ^3^ Gain = difference expressed in % between the root mean square difference between manufacturer’s and externally predicted values estimated before and after cleaning with the considered method. ^4^ N loss = the percentage of samples discarded using the tested cleaning procedure. ^5^ Gain:loss = the ratio of the GainRMSD to N loss.

## Data Availability

3rd Party Data.
